# The potential roles of T-type Ca^2+^ channels in motor coordination

**DOI:** 10.3389/fncir.2013.00172

**Published:** 2013-10-28

**Authors:** Young-Gyun Park, Jeongjin Kim, Daesoo Kim

**Affiliations:** ^1^Department of Biological Sciences, Korea Advanced Institute of Science and TechnologyDaejeon, South Korea; ^2^KAIST Institute for the BioCentury, Korea Advanced Institute of Science and TechnologyDaejeon, South Korea

**Keywords:** T-type Ca^2+^ channels, tremors, motor coordination, inferior olive, thalamocortical neurons

## Abstract

Specific behavioral patterns are expressed by complex combinations of muscle coordination. Tremors are simple behavioral patterns and are the focus of studies investigating motor coordination mechanisms in the brain. T-type Ca^2+^ channels mediate intrinsic neuronal oscillations and rhythmic burst spiking, and facilitate the generation of tremor rhythms in motor circuits. Despite substantial evidence that T-type Ca^2+^ channels mediate pathological tremors, their roles in physiological motor coordination and behavior remain unknown. Here, we review recent progress in understanding the roles that T-type Ca^2+^ channels play under pathological conditions, and discuss the potential relevance of these channels in mediating physiological motor coordination.

## Introduction

Unraveling the mechanisms that underlie specific motor patterns is a challenging issue in neuroscience. Motor coordination is a complex process that determines the timing and sequence of both activation and relaxation of a huge number of muscles (reviewed by Mauk et al., 2000; De Zeeuw et al., 2011). More than one gigahertz of information processing is required for the optimal execution of even the simplest motor behavior, such as holding a cup.

Tremors are one of the simplest forms of motor coordination and are characterized by involuntary rhythmic movements of either the whole body or of body parts (reviewed by Findley, 1995; Grimaldi and Manto, 2008). Low amplitude tremor, also known as physiological tremor, exist in humans and animals during normal states and may function to help behavioral control (Elble et al., 1984). High amplitude tremors that interfere with voluntary movements are observed in pathological conditions (reviewed by Deuschl et al., 2001). These pathological tremors are classified into different types, and are associated with specific neural mechanisms and circuits (Table 1) (reviewed by Wilms et al., 1999; Deuschl et al., 2001).

**Table 1:** **Mechanisms of pathological tremor**.

**Region**	**Tremor**	**Proposed mechanism**	**References**
Inferior olive (IO)	Essential tremor	Enhanced sub-threshold oscillation by potentiation of T-type Ca^2+^ channel	de Montigny and Lamarre, 1973; Hallett and Dubinsky, 1993; Wills et al., 1994; Louis et al., 2006; Park et al., 2010
	Symptomatic palatal tremor	Neuronal hypersynchrony	Klien, 1907; Lapresle, 1979; Deuschl et al., 2001
Purkinje cell	Essential tremor	Neuronal oscillation coherent with harmaline-induced tremor	Lamarre et al., 1971; Wills et al., 1994; Pinto et al., 2003; Axelrad et al., 2008
	Cerebellar tremor	Malfunction of the cerebellar feed-forward circuit	Elble et al., 1984; Thach et al., 1992
Deep cerebellar nuclei (DCN)	Essential tremor	Neuronal oscillation coherent with harmaline-induced tremor	Lamarre et al., 1971
	Cerebellar tremor	Malfunction of the cerebellar feed-forward circuit	Elble et al., 1984; Thach et al., 1992
Basal ganglia	Parkinson tremor	Dopamine deficiency in medial SNc and rhythmic burst firing in Gpe and STN	Guiot, 1958; Hassler et al., 1960; Bergman et al., 1990; Paulus and Jellinger, 1991; Brooks et al., 1992; Hirsch et al., 1992; Vitek et al., 1994; Jellinger, 1999; Deuschl et al., 2000
Ventral thalamus	Essential tremor	Neuronal oscillation coherent with harmaline-induced tremor	Jasper et al., 1970; Hallett and Dubinsky, 1993; Findley, 1996; Herzog et al., 2007
	Parkinson tremor	Transmission of tremor-related burst activity from basal ganglia to cortex	Guiot, 1958; Krack et al., 1999
Sensori-motor cortex	Essential tremor	Thalamocortical oscillations	Jasper et al., 1970; Halliday et al., 2000; Hellwig et al., 2001; Pinto et al., 2003; Kim et al., 2006; Miyagishima et al., 2007
	Parkinson tremor	Thalamocortical oscillations	Timmermann et al., 2002; Britton et al., 2004

**Figure 1:**
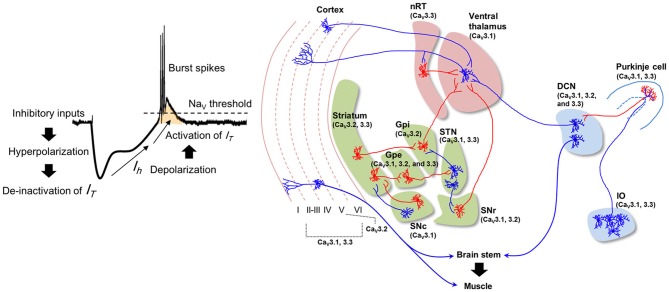
**T-type Ca^2+^ channels and pathological tremor pathways.** Potentiation of T-type Ca^2+^ conductance and the generation of low-threshold spikes (LTS) (*left*). Hyperpolarization and subsequent depolarization by HCN channel-mediated currents (*I*_*h*_) open T-type Ca^2+^ channels. *I*_*T*_: T-type calcium current; Na_V_: voltage-gated sodium channel. Neural pathways involved in pathological tremors (*right*). Blue indicates excitatory neurons, and red indicates inhibitory neurons. The blue regions represent the olivocerebellar pathway, the green regions represent basal ganglia circuits, and the red regions indicate thalamocortical pathways. The T-type Ca^2+^ channel isoforms (Ca_V_3.1, 3.2, and 3.3) expressed in these regions are indicated (Lein et al., 2006). IO: Inferior olive; DCN: deep cerebellar nuclei; SNc: substantia nigra compacta; Gpe: globus pallidus externa; Gpi: globus pallidus interna; STN: subthalamic nucleus; SNr: substantia nigra reticulata; nRT: nucleus reticularis of the thalamus.

T-type Ca^2+^ channels (Ca_V_3.1, 3.2, and 3.3) modulate both physiological and pathological rhythms in the brain (Crunelli et al., 1989; reviewed by Huguenard, 1996). These channels mediate the generation of low-threshold spikes (LTS) in response to hyperpolarizing membrane potentials elicited by inhibitory inputs. LTS regulate neural oscillations, resonance, and synchrony (Llinas and Yarom, 1986; Crunelli et al., 1989; Kim et al., 2001, 2011; Mangoni et al., 2006) (Figure 1, *left*). Pharmacological and genetic studies show that T-type Ca^2+^ channels are also involved in the generation of pathological tremors (Sinton et al., 1989; Handforth et al., 2010). However, the precise roles of T-type Ca^2+^ channels in physiological tremors and in normal motor coordination remain unknown. Here, we propose potential roles for T-type Ca^2+^ channels in physiological motor functions based on their pathological roles.

## Roles of T-type Ca^2+^ channels in generating pathological tremors

### T-type Ca^2+^ channels in essential tremor

Essential tremor is the most common form of movement disorder (Kurtzke, 1982; reviewed by Louis, 2005) and is characterized by postural and kinetic tremors at 4–12 Hz (Bain et al., 2000; Brennan et al., 2002). Harmaline is a plant-derived metabolite (Lutomski et al., 1974) that induces ET-like tremors and tremor-related neural oscillations in both humans and animals (Battista et al., 1969; de Montigny and Lamarre, 1973; Llinas and Volkind, 1973; Ahmed and Taylor, 2012). Since harmaline binds to various ion channels, including glutamate receptors, GABA receptors, and voltage-gated Ca^2+^ channels (Du et al., 1997; Glennon et al., 2000; Splettstoesser et al., 2005), specific molecular mechanism that underlies harmaline tremor have been unclear.

Inferior olive (IO) neurons are also implicated in the generation of harmaline tremor. IO lesions reduce harmaline tremors in rats (Simantov et al., 1976). Harmaline also alters the intrinsic properties of IO neurons: it increases LTS and amplifies sub-threshold oscillations (STOs). Both of these properties are dependent upon the conductance of T-type Ca^2+^ channels (Llinas and Yarom, 1986; Crunelli et al., 1989; Park et al., 2010). A non-selective T-type Ca^2+^ channel inhibitor, 1-octanol, reduces harmaline-induced tremors in rats (Sinton et al., 1989), supporting the role of T-type Ca^2+^ channels in harmaline-induced tremor.

There are three distinct isoforms of T-type Ca^2+^ channels, Ca_V_3.1, 3.2, and 3.3 (Cribbs et al., 1998; Perez-Reyes et al., 1998; Lee et al., 1999). Ca_V_3.1 is the major isoform expressed in the IO, Purkinje cells, and deep cerebellar nuclei (DCN) (Figure 1, *right*) (Talley et al., 1999; Lein et al., 2006). Ca_V_3.1^−/−^ mice treated with harmaline (dose: 9 mg/kg) do not display behavioral tremors, 4–10 Hz tremor-related oscillation in the olivocerebellar pathway, or STOs in IO neurons (Park et al., 2010). Patch clamp recording revealed that harmaline inhibits the activation of Ca_V_3.1 channels while also promoting their de-inactivation. These effects result in a net potentiation of Ca_V_3.1 channels under physiological conditions (Park et al., 2010).

Other ionic mechanisms and their interactions with Ca_V_3.1 channel could also contribute to the generation of harmaline tremor. A Ca^2+^ activated K^+^ channel isoform (KCa1.1) is known to form a complex with Ca_V_3 channels and be activated in response to Ca_V_3—mediated calcium influx (Rehak et al., 2013). Interaction between Ca_V_3 and Ca^2+^ activated K^+^ channels could possibly be involved in the generation of STO and its exaggeration during harmaline tremor. Hyperpolarization-activated cation channel is another candidate. Activation of this channel could contribute to slow rebound potential in STOs. This channel is also involved in rhythmic thalamic oscillation and the blockade of the channel ameliorates oscillation induced by hyperpolarizing current injection into IO neurons (Bal and McCormick, 1997). Investigation of harmaline tremor and tremor rhythm in Ca^2+^ activated K^+^ channel or hyperpolarization-activated cation channel knockout mice is required to test these possibilities.

GABA-A1 receptor knockout mice (a1^−/−^) are a genetic model of essential tremor. These mutant mice display ~25 Hz ET-like tremors (Kralic et al., 2005). A subset of non-selective T-type Ca^2+^ channel antagonists (ethosuximide, zonisamide, ECN, KYS05064, and NNC 55-0396) ameliorate both a1^−/−^ mouse tremors and harmaline tremor (Handforth et al., 2010). While Ca_V_3.1 knockout mice display reduced harmaline tremor (Park et al., 2010), double knockout mice (Ca_V_3.1^−/−^ and a1^−/−^) exhibit exacerbated tremor behavior (Chang et al., 2011). Thus, these two distinct animal models of essential tremor (a1^−/−^ and harmaline-induced tremors) likely result from different mechanisms, such as the involvement of distinct T-type Ca^2+^ channel isoforms (e.g., Harmaline tremor by Ca_V_3.1 and a1^−/−^ tremor by Ca_V_3.2 or 3.3). The heterogeneity of essential tremor is well-described by clinical studies (Kovach et al., 2001; Louis et al., 2007). Future studies are necessary to determine how the other T-type isoforms contribute to essential tremor.

### T-type Ca^2+^ channels in parkinson tremors

Resting tremor is one of the most detrimental symptoms experienced by Parkinson's disease (PD) patients. PD is caused by a dopamine deficiency in the brain. Rhythmic stimulation of the motor cortex via subcortical pathway is thought to underlie resting tremor of PD patients (Plenz and Kital, 1999; Magnin et al., 2000; Chan et al., 2011). There are several hypotheses about the origin of the tremor.

The basal ganglia circuit hypothesis states that rhythmic burst firings of neurons in subthalamic nuclei (STN) underlie the resting tremor in PD patients (Magnin et al., 2000; Chan et al., 2011). Suppressing STN activity using deep brain stimulation ameliorates resting tremor in PD patient (Kumar et al., 1998; Sturman et al., 2004; Amtage et al., 2008). STN burst activity is associated with the activation of T-type Ca^2+^ channels (Beurrier et al., 1999; Tai et al., 2011). Moreover, pharmacological inhibition of T-type Ca^2+^ channels in the STN rescues locomotor deficits in rat PD models, while effect on the resting tremor was not accessed (Tai et al., 2011). Isoforms of T-type Ca^2+^ channels that express in STN (Ca_V_3.1 and 3.3) (Figure 1) could possibly play a role in the generation of resting tremor in PD patients.

The ventrolateral (VL) thalamus is another candidate for PD resting tremor. Dopamine deficiency in PD would result in the activation of globus pallidus interna (Gpi) and substantia nigra reticulata (SNr) neurons that provides GABAergic input to the VL thalamus (Vitek, 2002). Subsequent hyperpolarization of the neurons may induce rhythmic LTS in VL thalamocortical (TC) neurons and thus generates the resting tremor. Consistently, tremor-related rhythmic LTS is observed in VL thalamic neurons (Zirh et al., 1998; Magnin et al., 2000; Pifl et al., 2012). These results suggest that Ca_V_3.1 expressing in VL thalamus (Figure 1) could be involved in the tremor generation. However, some other studies in PD patients also reports that LTS in VL neurons do not coincide with ongoing resting tremor (Zirh et al., 1998; Magnin et al., 2000). Thus, the role of thalamic burst firing in the resting tremor is still controversial.

A third hypothesis is the “dimmer-switch model” which states that core tremor activities are expressed by the cerebello-thalamo-cortical circuit (Figure 1, *right*) (Helmich et al., 2012). Hyperactivity in the cerebellum of PD patients is reported (Ghaemi et al., 2002; Timmermann et al., 2002), which is concomitant with the activation of sensory and motor cortices responsible for hand exhibiting resting tremor (Timmermann et al., 2004; Pollok et al., 2009). Deep brain stimulation of the STN or Gpi, or the administration of Levodopa normalizes cerebellar activity and improves tremor in PD patients (Payoux et al., 2008; Wu et al., 2009). This may be due to the ventral intermediate thalamus (which receives excitatory inputs from the cerebellum) acting as a critical relay station in the cerebello-thalamo-cortical circuit (Lenz et al., 1995; Tarsy et al., 2008). Consistently, PD resting tremor is suppressed by stimulation of the ventral intermediate thalamus, with decreased blood flow in cerebellar cortex (Deiber et al., 1993).

In spite of these evidences that T-type Ca^2+^ channels are involved in neuronal burst activity and oscillations in PD tremor circuits, there is no direct evidence that links T-type Ca^2+^ channels to resting tremor. One of the main obstacles on defining the role of T-type Ca^2+^ channels in PD tremor is a lack of robust rodent models that display resting tremor (Potashkin et al., 2010). Developing a robust resting tremor model and modulating T-type Ca^2+^ channels in the model might unravel the mechanism of PD resting tremor.

### T-type Ca^2+^ channels in palatal tremor

Palatal tremor, also called palatomyoclonus, is characterized by rhythmic movement of soft palate and sometimes of other muscles (Deuschl et al., 2001). Hypertrophy of IO neurons has been proposed as a pathologic substrate of the tremor (Deuschl et al., 2000; Pearce, 2008). This condition develops after lesions in the brainstem or cerebellum, manifesting as tremor in body parts contralateral to the region of damage in both human and animals (De Zeeuw et al., 1998; Deuschl et al., 2000). Studies of hemicerebellectomized animals reveal that hypertrophic IO neurons show failure in after-depolarization of action potential and have decreased numbers of GABAergic boutons in their dendrites (Ruigrok et al., 1990; De Zeeuw et al., 1998).

Because GABAergic input to IO neuron modulates electrotonic coupling of IO neurons (Sotelo et al., 1986; Leznik et al., 2002), it can be inferred that synchrony between IO neurons would be enhanced in the hypertrophic IO (Deuschl et al., 2000). This might entrain larger range of IO neurons with synchronized STOs, resulting rhythmic activations required for palatal tremor (Deuschl et al., 2000).

Ca_V_3.1 could also be involved in palatal tremor generation, in consideration of its role in STOs (Llinas and Yarom, 1986; Choi et al., 2010; Park et al., 2010). However, the contribution of Ca_V_3.1 would be different from the case of harmaline tremor. Potentiation of the Ca_V_3.1 channel is required for harmaline tremor (Park et al., 2010). Hyperpolarization of IO neurons contributes Ca_V_3.1 potentiation which amplifies STOs and subsequently generates tremor rhythm. (Park et al., 2010). In palatal tremor, however, hypertrophic IO neurons seem to be depolarized, meaning that Ca_V_3.1 might not be potentiated (Crunelli et al., 1989). Instead, increased firing rate with enhanced synchrony in IO neurons could, when combined with basal STOs generate a rhythmic activity for palatal tremor. Investigation of palatal tremor in Ca_V_3.1^−/−^ mice and changes in the Ca_V_3.1 activity may shed light on the differential mechanisms of IO-dependent tremor generation by T-type Ca^2+^ channel.

## The role of T-type Ca^2+^ channels in generating physiological motor functions

While their association with pathological tremors (Table 1) is well-understood, whether T-type Ca^2+^ channels contribute to physiological motor functions is unclear. Both Ca_V_3.1^−/−^ (Park et al., 2010) and Ca_V_3.2^−/−^ mice (Choi et al., 2006) do not have significant motor defects. In addition, overexpression of the *C*a_V_3.1 gene in mouse brain does not result in motor dysfunction (Ernst et al., 2009). These results might be due to compensatory expressions between Ca_V_3 channels, or the possibility that motor defects in Ca_V_3 mutants are not be able to be examined by conventional motor tests. Below, we summarize the potential roles of T-type Ca^2+^ channels in physiological motor behavior and describe how to study these potential roles.

### Generation of physiological tremors by T-type Ca^2+^ channels

Physiological tremors are induced by extrinsic factors such as gravity force (Marsden et al., 1969; Young and Hagbarth, 1980), or by central mechanisms such as a tremor rhythm pacemaker in the brain (Hagbarth et al., 1983; Vallbo and Wessberg, 1993; Llinas and Pare, 1994). 8–12 Hz component of physiological tremor is associated with central mechanisms because this component is unaffected by extrinsic factors (Elble and Randall, 1976; Vallbo and Wessberg, 1993).

The intrinsic rhythmicity of IO neurons may be one of these central mechanisms (de Montigny and Lamarre, 1973; Llinas and Pare, 1994; Findley, 1995), since the frequencies of STOs are around 10 Hz (Llinas and Yarom, 1986; Chorev et al., 2007; Khosrovani et al., 2007). These frequencies are similar to those of physiological tremors of humans and animals (Elble and Randall, 1976; Elble et al., 1984; Vallbo and Wessberg, 1993). Moreover, vibrissal movement generated at around 10 Hz (Fukuda et al., 1989) is abolished by electrolytic lesions of the IO in rats (Semba and Komisaruk, 1984), supporting that STOs in IO neurons could be the origin of the physiological tremor.

**Figure 2:**
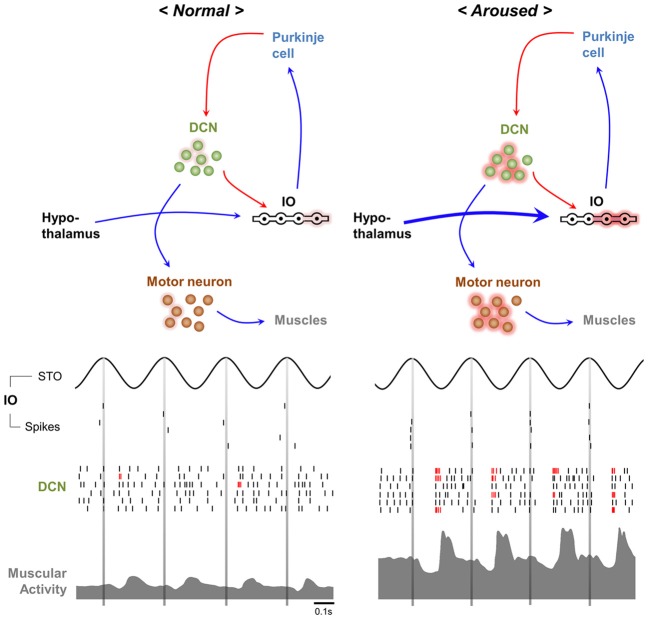
**Hypothetical mechanisms underlying normal and arousal-enhanced physiological tremors.** Physiological tremor generation (*left*). Adjacent IO neurons share ~10 Hz STOs via gap junctions, which results in ~10 Hz rhythms in the population of cells. Action potentials propagate from IO neurons to Purkinje cells and then to the DCN, thereby causing inhibitory responses and then rebound excitation in DCN neurons. The rhythmicity of the IO neuron population is converted into the rhythmicity of individual DCN neurons, which results in 10 Hz rhythmic muscle contractions. Increased excitatory inputs from hypothalamic neurons depolarize the membrane potentials of IO neurons upon arousal (*right*), which increases the ~10 Hz rhythmic LTS (red bars) in DCN neurons and muscle contractions.

While the frequencies of STOs are ~10 Hz, the average firing rate of IO neuron is about 1 Hz, suggesting that individual IO neurons are insufficient to generate signal for 10 Hz physiological tremor (Keating and Thach, 1997; Lang et al., 1999; Chorev et al., 2007). However, presence of gap junction in IO and property of their connectivity with descending motor pathway support that STOs in IO neurons could be responsible for physiological tremor. Gap junctions synchronize IO neurons with a 10 Hz STO rhythm (Long et al., 2002; Van Der Giessen et al., 2008), ensuring that some IO neurons fire with the 10 Hz cycle at the population level (Chorev et al., 2007; Park et al., 2010). Since multiple IO neurons innervate a DCN neuron via Purkinje cells (Van der Want et al., 2004), an individual DCN neuron may receive 10Hz rhythmic input, as well as pharmacologically induced synchronization of IO rhythmic activity evoke rhythmic modulation of DCN firing with same frequency (Lamarre et al., 1971; Park et al., 2010). The 10 Hz oscillation in DCN may recruit brainstem nuclei (e.g., the red nucleus and the lateral reticular formation) and motor neurons, resulting in 10 Hz physiological tremor (Figure 2, *left*).

One simple way to link STOs and physiological tremor would be to examine physiological tremors in Ca_V_3.1^−/−^ mice that lack STOs of IO neurons (Park et al., 2010). Unfortunately, physiological tremors are not well documented in mice. One study reported that 20–35 Hz forelimb vibration may reflect physiological tremor in mice (Kralic et al., 2005). However, the 20–35 Hz vibration could be an artifact of resonant frequencies in the recording system of the study. Application of more sensitive techniques, such as electromagnetic or optoelectronic detection methods (Grimaldi and Manto, 2008), might be necessary for future studies on the mechanism of physiological tremors in mice.

### Arousal-induced enhancement of physiological tremors

Physiological tremors in both humans and animals are amplified in response to various alerting stimuli such as anger, novelty, or stress (Günther et al., 1983; Duan et al., 1996; Klein, 2002; Siniscalchi et al., 2013). The enhanced physiological tremor is probably important for providing optimal muscular coordination during arousal. Because the hypothalamus controls arousal (Lin et al., 1988; Adamantidis et al., 2007; Tsunematsu et al., 2011) and its projections to IO neurons are revealed by anterograde tracing with AAV virus (Lein et al., 2006), we here propose that arousal-induced physiological tremors could be dependent upon the excitation of IO neurons (Figure 2, *right*) by hypothalamic input.

Increased excitatory input to the IO may enhance synchrony between IO neurons, since infusion of a glutamate receptor antagonist into the IO decreases complex spike synchrony in the mediolateral direction (Lang, 2002). Otherwise, the increased firing rate of IO neurons might also raises the probability of synchronous firing among IO neurons. Hypersynchronous IO firing would be translated into rhythmic LTS in individual DCN neurons (Hoebeek et al., 2010; De Zeeuw et al., 2011) that might cause high amplitude physiological tremor during arousal (Witter et al., 2013) (Figure 2, *right*).

Among the three isoforms of T-type Ca^2+^ channels expressed in DCN neurons (Figure 1), Ca_V_3.1 is thought to serve a role in the tremor, as Ca_V_3.1 is responsible for generating LTS with multiple sodium spikes in DCN neurons (Molineux et al., 2006). Analysis of muscular activities of Ca_V_3.1^−/−^ mice in response to novel contexts is required to address this possibility.

### Modulation of movement initiation timing

**Figure 3:**
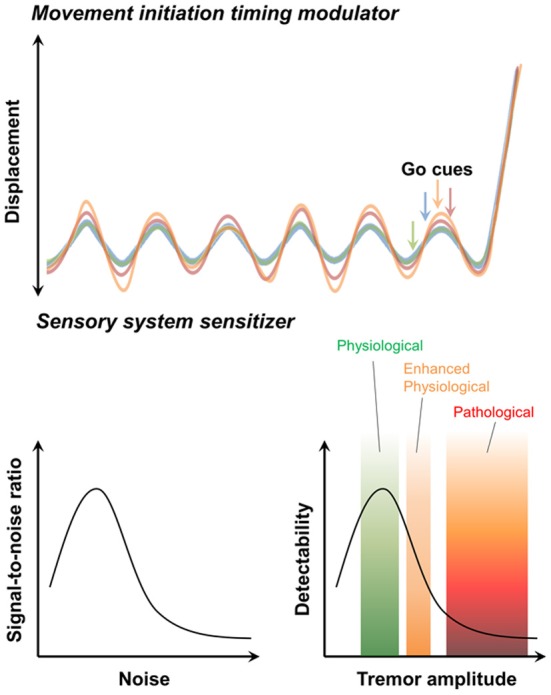
**Possible contribution of physiological tremors to motor function.** The movement initiation timing modulator hypothesis (*upper*). Initiation of voluntary movement in response to “Go” cues depends on the phase of the ongoing physiological tremor. The sensory system sensitivity hypothesis (*lower*). The stochastic resonance signature (*left*). “Noise” caused by physiological tremors increases the ability to detect signals. By contrast, pathological tremors decrease the ability of the sensory system to detect signals (*right*).

In humans movement initiation is connected to the timing of physiological tremor in some respects (Travis, 1929; Goodman and Kelso, 1983). For example, the 10 Hz periodicity of motor initiation timing is observed in various parts of human body (Harter and White, 1968). While this restriction may reduce the temporal precision of movement initiation (Figure 3, upper) (Lakie and Combes, 2000; Lakie, 2010), physiological tremor also helps overcome muscular friction prior to action initiation and permits more powerful and faster movements (Adamovich et al., 1994; de Rugy and Sternad, 2003). Modulation of Cav3.1 in IO neurons hypothesized to be involved in physiological tremor and is expected to affect the kinetics of movement inhibition.

The basal ganglia-thalamocortical circuit is important for movement initiation in both rodents and primates (Yin and Knowlton, 2006; Bédard and Sanes, 2011). Studies show that the GABAergic outputs of medium spiny neurons expressing dopamine receptor 1 or 2 in the dorsal striatum play inhibitory or facilitatory roles, respectively, in movement initiation (Kravitz and Kreitzer, 2012). However, *in vivo* imaging study shows that the both types of medium spiny neurons are activated during movement initiation in mice (Cui et al., 2013). Therefore, the role of medium spiny neurons in movement initiation is still controversial (Surmeier, 2013).

During resting states, there is increased inhibition of VL neurons by the basal ganglia, which in turn raises the threshold for the onset of movement initiation signals in the thalamocortical pathway. VL neurons also receive excitatory signals from DCN through monosynaptic connections between them (Shinoda et al., 1985, 1993; Lein et al., 2006). Consistent with their supposed role in generating 10 Hz DCN rhythms (Figure 2, *left*), T-type Ca^2+^ channels may also play a role in movement initiation. 10 Hz rhythmic signals in DCN neurons may reduce the action potential threshold in VL neurons. It will be necessary to selectively inhibit DCN-VL circuits in future studies.

### Physiological tremors provide preparation for emergent motor responses

Emergent motor responses are critical for the survival of animals in nature. The unexpectancy hypothesis of IO function (Devor, 2002) states that IO neurons reliably respond to unexpected motor disturbances. One example is the increased IO neuronal excitability when cat misses a step on a ladder by unexpected rung down (Andersson and Armstrong, 1987). Consistent with this idea, IO neuronal excitability decreases after rodents learn the timing of air puffs (Kim et al., 1998). As with hypothalamic control of the IO, unexpected external stimuli could activate hypothalamic arousal pathways and amplify 10 Hz physiological tremors through the action of Ca_V_3.1 in the DCN (Figure 2, *right*). This enhanced tremor may help overcome inertial resistances and synchronize muscles when emergent motor responses are required (Greene, 1972).

Meanwhile, T-type Ca^2+^ channel also could contribute to the generation of emergent movement through a DCN-dependent mechanism. LTS in DCN neurons is proposed to provide synchronous and strong output to descending motor pathways (De Zeeuw et al., 2011). Recently, a study with optogenetic modulation of Purkinje cells reveals that induced LTS in DCN neurons can evoke emergent movement (Witter et al., 2013). Cessation of induced Purkinje cell ensemble activity induces rebound activity in DCN and timed movement whose amplitude is dependent on the degree of Purkinje cell activation. As Ca_V_3.1 is responsible for generating LTS with multiple sodium spikes in DCN neurons (Molineux et al., 2006), analysis of emergent motor responses generated by Ca_V_3.1^−/−^ mice would help to access this idea.

### Sensory sensitization hypothesis

Studies of human sensory perception suggest that physiological tremors can facilitate sensory functions. For example, eyeball tremors sensitize visual function (Hennig et al., 2002). Intentional suppression of physiological limb tremors reduces visual cue-tracking abilities (Daneault et al., 2011). Moreover, artificial vibrations of foot muscles, which could mimic sensory feedback by physiological tremors, increase somatosensory sensitivity (Liu et al., 2002).

Common sense suggests that proprioceptive feedback signals resulted from physiological tremors act as “noise” that might interfere with sensation. However, the stochastic resonance theory (McNamara et al., 1988; Wiesenfeld and Moss, 1995) states that moderate levels of “noise” actually facilitate signal detection in nervous system (Figure 3, *lower*) (Douglass et al., 1993; Levin and Miller, 1996). Therefore, by providing moderate noise, physiological tremors could enhance sensory detection (Figure 3, *lower*) and subsequently raise motor performances. The role of Ca_V_3.1 in the IO in generating physiological tremor would be critical in this process.

T-type Ca^2+^ channels in the thalamus might also be associated with sensory sensitization. VL thalamocortical relay neurons may receive tremor signals generated by the cerebellum through DCN-VL connections. Rhythmic activation of the VL neurons activates GABAergic nRT neurons through reciprocal connections between TC and nRT neurons (Huguenard and Prince, 1994). Activation of nRT neurons can in turn induces rhythmic inhibition and thus LTS in VL thalamic neurons, while non-specifically inhibit TC neurons with other modalities. This may increase sensory coding in TC neurons: intensifying VL thalamic input with LTS while filtering out weak intensity sensory stimuli from other thalamic nuclei (Pinault and Deschênes, 2001). The role of LTS in sensory processing is controversial (Beurrier et al., 1999; Perez-Reyes, 2003), and studies evaluating sensory processing in the knockout mice of Ca_V_3.1 knockout mice would verify this hypothesis.

### Muscular activity changes according to emotion

Emotions play an essential role in modulating motor functions. Anger or panic increases physiological tremor (Duan et al., 1996; Klein, 2002; Siniscalchi et al., 2013) and can severely impair motor coordination in human (Parker et al., 1993; Allgulander et al., 2003). In the patient with dystonia which is characterized by sustained synchronous muscle contractions and twisting body parts (Herz, 1944), dystonic symptoms become exaggerated in response to fear or stress (Burgyone et al., 2004; Jabusch and Altenmuller, 2004; Calderon et al., 2011)

The hypothalamus is activated by either fear or stress (Porter, 1952; Yokoo et al., 1990; Tsunematsu et al., 2011) and connections between the hypothalamus and the IO (Lein et al., 2006) lead some to speculate that the activation of IO neurons may mediate increased dystonic responses as a result of fear or stress. Subsequent activation of LTS in DCN neurons by synchronous inputs from IO neurons may increase muscular synchrony and symptoms of dystonia (Figure 2, *right*). Because Ca_V_3.1 channel majorly generate LTS in DCN neurons, knockout of Ca_V_3.1, or the application of T-type Ca^2+^ channel blockers in the DCN may ameliorate emotion-dependent motor symptoms.

## Conclusion

T-type Ca^2+^ channels are expressed in neurons that comprise motor circuits, but the roles of these channels in physiological motor functions remain unknown. Because T-type Ca^2+^ channels are involved in generating pathological tremors, we propose that these channels may also play important roles in various physiological motor functions by enhancing physiological tremors or muscle tone. Optogenetic techniques (Boyden et al., 2005; Deisseroth, 2010) may be useful for identifying the neural circuits and cell types that underlie each of these physiological motor functions. Once the neural circuits are defined, then isoform-specific knockdowns of T-type Ca^2+^ channels (Park et al., 2010) can be applied to identified circuits. Future studies into physiological tremors and T-type Ca^2+^ channels using advanced technologies will improve our understanding of the neural mechanisms underlying higher motor coordination.

### Conflict of interest statement

The authors declare that the research was conducted in the absence of any commercial or financial relationships that could be construed as a potential conflict of interest.
